# Association between number of remaining teeth and healthy aging in Japanese older people: The Ohsaki Cohort 2006 Study

**DOI:** 10.1111/ggi.14320

**Published:** 2021-12-01

**Authors:** Sanae Matsuyama, Yukai Lu, Jun Aida, Fumiya Tanji, Ichiro Tsuji

**Affiliations:** ^1^ Division of Epidemiology, Department of Health Informatics and Public Health School of Public Health, Tohoku University Graduate School of Medicine Sendai Japan; ^2^ Department of Oral Health Promotion Graduate School of Medical and Dental Sciences, Tokyo Medical and Dental University Tokyo Japan; ^3^ Faculty of Nursing Japanese Red Cross Akita College of Nursing Akita Japan

**Keywords:** healthy aging, longitudinal study, oral health, remaining teeth, successful aging

## Abstract

**Aim:**

Maintaining ≥20 teeth is a public health goal worldwide. Healthy aging, which includes psychological and social well‐being, as well as physical indicators, has attracted a great deal of attention with the progression of aging societies. However, no studies have examined the association between the number of remaining teeth and healthy aging. This study aimed to investigate the association between the number of remaining teeth and healthy aging.

**Methods:**

This community‐based longitudinal cohort study included 8300 Japanese people aged ≥65 years who were free of disability and depression in the baseline survey in 2006. The participants were categorized into four groups according to the number of remaining teeth at baseline: 0–9, 10–19, 20–24 and ≥25. The primary outcome was healthy aging (defined as meeting all four of the following criteria: free of disability, free of depression, high health‐related quality of life and high life satisfaction), as assessed by a questionnaire survey carried out in 2017. Multiple logistic regression was used to calculate the corresponding odds ratios and 95% confidence intervals.

**Results:**

During about 11 years of follow‐up, 621 (7.5%) participants attained healthy aging. Participants with ≥20 remaining teeth showed a higher healthy aging rate. Compared with participants with 0–9 teeth, the multivariate‐adjusted odds ratios (95% confidence intervals) for 10–19, 20–24 and ≥25 teeth were 0.98 (0.77–1.26), 1.28 (1.01–1.63) and 1.59 (1.24–2.03), respectively.

**Conclusions:**

These findings suggest that maintaining ≥20 teeth was associated with healthy aging. **Geriatr Gerontol Int 2022; 22: 68–74**.

## Introduction

With population aging, “successful aging” or “healthy aging” has attracted a great deal of attention worldwide, because people have begun to seek well‐being during aging rather than mere longevity and absence of disability.^1^, ^2^

The concept of “successful aging” was first proposed by Rowe and Kahn, and they defined successful aging as, “low probability of disease and disease‐related disability and related risk factors,” “high cognitive and physical functional capacity” and “active engagement with life”.^3^, ^4^ The concept of successful aging has been further developed, and the World Health Organization in 2015 began using the term “healthy aging,” defined as “the process of developing and maintaining the functional ability that enables well‐being in older age”.^5^ This definition recognizes the enhanced importance of promoting well‐being among the aged, which includes domains, such as happiness, satisfaction and fulfillment.^5^ Thus, our definition of healthy aging, included not only physiological indicators (i.e. free of disability), but also factors regarding psychological and social well‐being (i.e. free of depression, high health‐related quality of life [HRQOL] and high life satisfaction).^1^

Good oral health has been shown to be associated with better physical or cognitive function.^6^, ^7^ In addition, systematic reviews have reported that maintaining natural teeth is associated with decreased risks of dementia^8^ and depression.^9^ The World Health Organization reported that oral health is an important component of general health and quality of life,^10^ and the goal of increasing the percentage of individuals with functional dentition (i.e. ≥21 remaining teeth) was advocated in Global Goals for Oral Health 2020.^11^ Furthermore, the goal of Health Japan 21, the national health promotion strategy implemented by the Ministry of Health, Labour and Welfare of Japan, is to increase the percentage of individuals maintaining ≥20 remaining teeth until the age of 80 years from 25% in 2005 to 50% by 2022.^12^ However, there is no clear evidence that ≥20 remaining teeth are required for good health. Additionally, to our knowledge, no studies have examined the association between number of remaining teeth and healthy aging.

Therefore, the present study aimed to investigate the association between number of remaining teeth and healthy aging using data from a community‐based longitudinal cohort study on older people in Japan. We defined healthy aging as not only physical capability, but also a broad domain of well‐being, including free of depression, high HRQOL and high life satisfaction.

## Methods

### Study participants

#### Baseline survey

**Figure 1:**
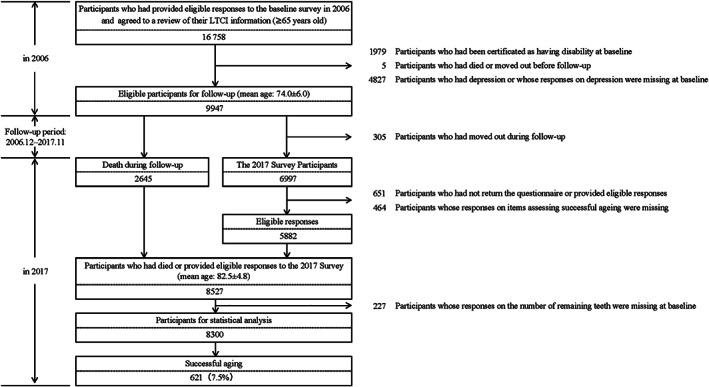
Flowchart of study participants. LTCI, long‐term care insurance.

The design of the Ohsaki Cohort 2006 Study has been described in detail elsewhere.^13^ In brief, the source population was composed of all citizens aged ≥65 years living in Ohsaki city in northeastern Japan (*n* = 31 694). The baseline survey was carried out between 1 and 15 December 2006. A questionnaire was distributed to individual households by the heads of individual administrative districts and then collected by mail. Among the source population, 23 091 persons who provided valid responses formed the study cohort. We further excluded 6333 people who did not provide written consent for review of their long‐term care insurance (LTCI) information, 1979 who had already been certified as having a disability by the LTCI system before follow up, five who had died or moved out of the district before follow up and 4827 who had depressive symptoms or whose responses regarding depression were missing at baseline. Finally, 9947 participants were followed from 16 December 2006 to 30 November 2017 (Fig. 1).

#### Healthy aging assessment survey (2017 Survey)

During the follow‐up period, 2645 participants died and 305 were lost because of moving out of the study area. Among the remaining 6997 survivors, we carried out a health‐related questionnaire survey in November 2017 (2017 Survey). The questionnaire included self‐report questions regarding depression, HRQOL and life satisfaction, which were the components used to assess healthy aging. We further excluded 651 participants who did not return a questionnaire or provide eligible responses, 464 whose responses on components assessing healthy aging were missing, and 227 whose answers on the number of remaining teeth were missing at baseline. Finally, 8300 participants, consisting of 2645 who died during follow up and 5655 who provided valid responses in the 2017 Survey, were included in the statistical analysis (Fig. 1).

#### Number of remaining teeth (exposure)

In the baseline questionnaire, we asked the respondents to classify their number of remaining teeth into the following six categories: all (28 teeth), most (25–27 teeth), moderate (20–24 teeth), about half (10–19 teeth), few (1–9 teeth) and none (0 teeth). Then, we divided the respondents into the following four groups according to quartiles of the categorization of the number of remaining teeth: 0–9 teeth, 10–19 teeth, 20–24 teeth and ≥25 teeth.

We also asked the respondents whether they used dentures. Then, we combined the responses regarding number of teeth and denture use, and divided the respondents into the following six groups: 0–9 teeth, 10–19 teeth without dentures, 10–19 teeth with dentures, 20–24 teeth without dentures, 20–24 teeth with dentures and ≥25 teeth. The respondents who answered 0–9 teeth and ≥25 teeth were not classified based on whether they used dentures, because those who had 0–9 teeth mostly did (94.0%), and those who had ≥25 teeth mostly did not (86.1%).

### Healthy aging (outcome)

#### Definition of healthy aging

The primary outcome of the present study was healthy aging, which consisted of the following four components according to our previous study^1^: (i) free of functional disability; (ii) free of depression; (iii) high HRQOL; and (iv) high life satisfaction. Only participants who met all four criteria were considered to have healthy aging. Participants who did not meet all four criteria were categorized as having normal aging, even if they had missing values for some of the components.

#### Functional disability ascertainment

In the present study, we defined incident functional disability as certification under the LTCI system in Japan (Support Level 1 or higher), which uses a nationally uniform standard of functional disability.^14^, ^15^ LTCI in Japan is a mandatory social insurance system that is meant to help frail older individuals carry out activities of daily living. LTCI certification was found to be associated with the ability to carry out activities of daily living in a community‐based study, and has previously been used as a measure of incident functional disability among older individuals in epidemiological studies.^16^ Data regarding incident functional disability, death or emigration during follow up were transferred from the Ohsaki City Government under the agreement on Epidemiologic Research and Privacy Protection.

#### Measurement of depression

Depression was measured both at baseline and in the 2017 Survey through the Depression and Suicide Screen. The Depression and Suicide Screen, which was developed in Japanese by Fujisawa *et al*., is a brief screening instrument for depression and suicidal ideation for the aged.^17^ This instrument is composed of the following five items: (1) “Is your life pretty full?”; (2) “Do you still enjoy doing the things you used to do?”; (3) “Do you think it is too much trouble to do the things you used to do?”; (4) “Do you feel that you are a useful person who is needed by others?”; and (5) “Do you feel tired without any specific reason?”. For items 1, 2) and 4, responses of “yes” are scored 0 and responses of “no” are scored 1, whereas for items 3 and 5, responses of “yes” are scored 1 and responses of “no” are scored 0. The cut‐off value of 1 (≤1 *vs* >1) produced satisfactory sensitivity and specificity in detecting depression (i.e. 70.5% and 72.9%, respectively), so we defined a Depression and Suicide Screen score of <2 as “free of depression”.^17^

#### Measurement of HRQOL

HRQOL was evaluated using the three‐level version of the European Quality of Life‐5 Dimensions (EQ‐5D‐3L) in the 2017 Survey.^18^ The EQ‐5D‐3L consists of the following five dimensions: (1) mobility; (2) self‐care; (3) usual activities; (4) pain/discomfort; and (5) anxiety/depression. The Japanese version of the EQ‐5D‐3L was developed by Tsuchiya *et al*.^19^ The results were coded and converted to a utility value score ranging from −0.111 to 1.000. An EQ‐5D‐3L score of 1.000 represents a state of full health; we defined this score as “high HRQOL”.

#### Measurement of life satisfaction

Life satisfaction was assessed in the 2017 Survey using the Satisfaction With Life Scale developed by Diener *et al*.^20^; we used the Japanese version, the reliability and validity of which were verified by Sumino.^21^ The Satisfaction With Life Scale is composed of five items, each of which is scored from 1 to 7 to indicate agreement, with a possible range from 5 (low satisfaction) to 35 (high satisfaction). We defined an Satisfaction With Life Scale score of ≥25 as “high life satisfaction”.

#### Covariates

Body mass index was calculated as the self‐reported body weight (kg) divided by the square of the self‐reported body height (m^2^).

The Kihon Checklist was developed by the Ministry of Health, Labour and Welfare of Japan to predict functional decline in community‐dwelling older individuals. With regard to the cognitive function score in the Kihon Checklist, respondents were asked about their current cognitive function status using three binary questions, yielding a total score ranging from 0 to 3 points. The validity of the cognitive function score in the Kihon Checklist was confirmed in a previous study using the Clinical Dementia Rating as the gold standard.^22^ According to previous studies, we classified individuals with a score of 0 as having better cognitive function.^22^

#### Statistical analysis

The participants' baseline characteristics were evaluated using the χ^2^‐test for variables of proportion and one‐factor analysis of variance for continuous variables. We used these methods to compare variables among groups with varying numbers of teeth.

Next, we used multiple logistic regression models to calculate the odds ratios (ORs) and 95% confidence intervals (CIs). Participants having 0–9 teeth were used as a reference category. Dummy variables were created for each multicategorical covariate for the corresponding model. For cases where values for a covariate were missing, we created a separate category for missing values and included this in the model. The following model was used to analyze the association between number of remaining teeth and healthy aging. The model was adjusted for sex, age, smoking status (current, former, never, or missing), drinking status (current, former, never, or missing), time spent walking (≥1, 0.5–1, or <0.5 h/day or missing), sleep duration (≤6, 7–8, or ≥9 h/day, or missing), education level (≤15, 16–18 or ≥19 years, or missing), history of disease (stroke, hypertension, myocardial infarction, cancer, or diabetes [yes or no for each term]), cognitive function score (0, ≥1 or missing) and social participation (volunteering, hobby activities, or activities in neighborhood association [yes or no for each term]), because the above variables were associated with health status.

We also carried out two sets of sensitivity analyses. First, we replaced the exposure by combining the number of teeth and whether they used dentures. Second, we replaced the outcome with survival and four components of healthy aging using the same multiple logistical regression model, respectively.

All statistical analyses were carried out using the sas software package (version 9.4; SAS Institute, Cary, NC, USA). All statistical tests were two‐sided, and *P*‐values <0.05 were considered significant.

#### Ethical issues

We considered the return of a completed questionnaire to imply consent to participate in the study involving the baseline survey data, subsequent follow up for death and emigration, and the 2017 Survey data. The Ethics Committee of the Tohoku University Graduate School of Medicine (Sendai, Japan) reviewed and approved the study protocols (Ohsaki Cohort 2006 Study: 2006–206; 2017 Survey: 2017‐1‐631).

## Results

In the present study, 8300 participants (48.3% men; mean [standard deviation] age at baseline in 2006, 73.3 [5.7] years) were included in the statistical analysis. During approximately 11 years of follow up, 621 (7.5%) participants met all the criteria for healthy aging, and 5746 (69.2%) survived.

Table 1 shows the characteristics of the study participants according to the number of remaining teeth. Those who had fewer teeth showed lower rate of healthy aging, were older, were likely to be women, were current smokers, spent less time walking, slept longer and had a lower education level.

**Table 1:** Baseline characteristics of the study participants according to number of remaining teeth (*n* = 8300)

	No. remaining teeth				*P*‐values
	0–9	10–19	20–24	≥25	
	(*n* = 3414)	(*n* = 2017)	(*n* = 1652)	(*n* = 1217)	
No. healthy aging (%)	154 (4.5)	131 (6.5)	171 (10.4)	165 (13.6)	<0.001
Mean age, years (SD)	75.4 (5.9)	72.8 (5.3)	71.5 (4.8)	70.8 (4.7)	<0.001
Sex (%)					
Men	44.9	48.6	52.5	51.9	<0.001
Women	55.1	51.4	47.5	48.2	
Body mass index, kg/m^2^ (%)					
≤18.4	5.3	3.9	2.5	3.7	<0.001
18.5–24.9	66.4	66.3	64.7	63.3	
≥25.0	28.3	29.8	32.8	33.0	
Smoking status (%)					
Current	15.1	14.6	11.5	9.3	<0.001
Former	28.6	27.8	27.7	26.9	
Never	56.3	57.6	60.8	63.8	
Drinking status (%)					
Current	34.8	43.0	49.1	48.5	<0.001
Former	11.0	10.2	7.5	9.1	
Never	54.2	46.8	43.4	42.4	
Time spent walking, h/day (%)					
≥1.0	29.1	31.7	31.4	31.7	<0.001
0.5–1.0	36.5	38.9	40.4	43.5	
<0.5	34.4	29.4	28.2	24.8	
Sleep duration, h/day (%)					
≤6	17.5	19.6	19.9	19.8	<0.001
7–8	58.5	61.0	63.0	65.3	
≥9	24.0	19.4	17.1	14.9	
Education level^†^ (%)					
≤15 years	31.4	25.1	23.9	18.7	<0.001
16–18 years	43.7	43.7	44.5	45.8	
≥19 years	24.9	31.2	31.6	35.5	
History of disease (%)					
Stroke	2.2	2.5	2.2	2.1	0.785
Hypertension	42.4	42.8	43.6	42.2	0.820
Myocardial infarction	4.9	4.1	3.4	3.4	0.026
Diabetes mellitus	11.7	11.0	9.4	10.3	0.091
Cancer	8.4	8.1	8.1	8.1	0.963
Better cognitive function^‡^ (%)	67.4	72.0	75.5	75.7	<0.001
Social participation (%)					
Volunteering	31.3	39.2	44.8	43.0	<0.001
Hobby activities	46.3	54.9	61.8	61.4	<0.001
Activities in neighborhood association	49.5	55.9	59.8	59.0	<0.001

^†^ Age at last school graduation.

^‡^ Cognitive function score in Kihon Checklist <1.

We used the χ^2^‐test for variables of proportion and one‐factor anova for continuous variables (missing value excluded).

Table S1 shows the characteristics of the study participants according to healthy aging or non‐healthy aging. Those who attained healthy aging were younger, likely to have a higher education level, had better cognitive function and has a higher rate of social participation.

Table 2 shows the association between the number of remaining teeth and healthy aging. Participants with ≥20 remaining teeth showed higher rates for healthy aging. Compared with participants with 0–9 teeth, the multivariate‐adjusted ORs (95% CIs) were 0.98 (0.77–1.26) for 10–19 teeth, 1.28 (1.01–1.63) for 20–24 teeth and 1.59 (1.24–2.03) for ≥25 teeth. The ORs for healthy aging did not increase linearly with the number of remaining teeth, rather it became significant for those having ≥20 teeth.

**Table 2:** Association between the number of remaining teeth and healthy aging (*n* = 8300)

	No. remaining teeth			
	0–9	10–19	20–24	≥25
	(*n* = 3414)	(*n* = 2017)	(*n* = 1652)	(*n* = 1217)
		OR (95% CI)	OR (95% CI)	OR (95% CI)
No. healthy aging (%)	154 (4.5)	131 (6.5)	171 (10.4)	165 (13.6)
Model^†^	1.00 (Ref.)	0.98 (0.77–1.26)	1.28 (1.01–1.63)	1.59 (1.24–2.03)

^†^ Adjustment items: sex, age (65–69, 70–74, 75–79, 80–84 or ≥85 years), smoking status (current, former, never or missing), drinking status (current, former, never or missing), time spent walking (≥1, 0.5–1 or <0.5 h/day, or missing), sleep duration (≤6, 7–8, or ≥9 h/day or missing), education level (≤15, 16–18 or ≥19 years, or missing), history of disease (stroke, hypertension, myocardial infarction, cancer or diabetes [yes or no for each term]), cognitive function score (0, ≥1 or missing) and social participation (volunteering, hobby activities or activities in neighborhood association [yes or no for each term]).

CI, confidence interval; OR, odds ratio; Ref., referent values.

This relationship did not differ when exposure was replaced with the combined number of teeth and use of dentures. The multivariate‐adjusted ORs (95% CIs) for 10–19 teeth without dentures, 10–19 teeth with dentures, 20–24 teeth without dentures, 20–24 teeth with dentures and ≥25 teeth were 0.97 (0.65–1.47), 0.98 (0.75–1.28), 1.18 (0.90–1.56), 1.46 (1.06–2.00) and 1.58 (1.23–2.03), respectively (Table 3).

**Table 3:** Association between the number of remaining teeth and without/with dentures and healthy aging (*n* = 8220)

	No. remaining teeth and use of dentures					
	0–9^†^	10–19		20–24		≥25^†^
		without dentures	with dentures	without dentures	with dentures	
	(*n* = 3404)	(*n* = 481)	(*n* = 1523)	(*n* = 1017)	(*n* = 606)	(*n* = 1189)
		OR (95% CI)	OR (95% CI)	OR (95% CI)	OR (95% CI)	OR (95% CI)
No. healthy aging (%)	154 (4.5)	31 (6.4)	99 (6.5)	102 (10.0)	67 (11.1)	162 (13.6)
Model^‡^	1.00 (Ref.)	0.97 (0.65–1.47)	0.98 (0.75–1.28)	1.18 (0.90–1.56)	1.46 (1.06–2.00)	1.58 (1.23–2.03)

^†^ The respondents who answered 0–9 teeth and ≥25 teeth were not classified based on whether they used dentures, because those who had 0–9 teeth mostly did (94.0%), and those who had ≥25 teeth mostly did not (86.1%).

^‡^ Adjustment items: sex, age (65–69, 70–74, 75–79, 80–84 or ≥85 years), smoking status (current, former, never or missing), drinking status (current, former, never or missing), time spent walking (≥1, 0.5–1 or <0.5 h/day, or missing), sleep duration (≤6, 7–8 or ≥9 h/day, or missing), education level (≤15, 16–18 or ≥19 years, or missing), history of disease (stroke, hypertension, myocardial infarction, cancer or diabetes [yes or no for each term]), cognitive function score (0, ≥1 or missing) and social participation (volunteering, hobby activities or activities in neighborhood association [yes or no for each term]).

CI, confidence interval; OR, odds ratio; Ref., referent values.

Table 4 shows the association between the number of remaining teeth and each component of healthy aging (survival, free of disability, free of depression, high HRQOL and high life satisfaction). Participants with ≥20 remaining teeth showed higher rates for each component of healthy aging; the corresponding multivariate‐adjusted ORs (95% CIs) for 10–19 teeth, 20–24 teeth and ≥25 teeth were 0.98 (0.77–1.26), 1.28 (1.01–1.63) and 1.59 (1.24–2.03), respectively, in survival; 0.95 (0.74–1.22), 1.20 (0.94–1.53) and 1.44 (1.12–1.85), respectively, in free of disability; 0.92 (0.71–1.19), 1.13 (0.88–1.44) and 1.31 (1.02–1.70), respectively, in free of depression; 0.92 (0.71–1.19), 1.14 (0.89–1.46) and 1.37 (1.06–1.77), respectively, in high HRQOL; and 0.93 (0.72–1.20), 1.14 (0.89–1.47) and 1.35 (1.05–1.75), respectively, in high life satisfaction.

**Table 4:** Association between the number of remaining teeth and survival and four components of healthy aging

	No. remaining teeth			
	0–9	10–19	20–24	≥25
		OR (95% CI)	OR (95% CI)	OR (95% CI)
Survival				
Percentages of participants that survived	60.5	70.0	77.2	81.7
Model^†^	1.00 (Ref.)	0.98 (0.77–1.26)	1.28 (1.01–1.63)	1.59 (1.24–2.03)
Free of disability				
Percentages of participants free of disability	56.0	66.6	74.2	76.5
Model^†^	1.00 (Ref.)	0.95 (0.74–1.22)	1.20 (0.94–1.53)	1.44 (1.12–1.85)
Free of depression				
Percentages of participants free of depression	47.5	52.8	61.1	66.5
Model^†^	1.00 (Ref.)	0.92 (0.71–1.19)	1.13 (0.88–1.44)	1.31 (1.02–1.70)
High HRQOL				
Percentages of participants with high HRQOL	24.2	27.9	33.5	39.8
Model^†^	1.00 (Ref.)	0.92 (0.71–1.19)	1.14 (0.89–1.46)	1.37 (1.06–1.77)
High life satisfaction				
Percentages of participants with high life satisfaction	35.3	36.6	42.0	42.3
Model^†^	1.00 (Ref.)	0.93 (0.72–1.20)	1.14 (0.89–1.47)	1.35 (1.05–1.75)

^†^ Adjustment items: sex, age (65–69, 70–74, 75–79, 80–84 or ≥85 years), smoking status (current, former, never or missing), drinking status (current, former, never or missing), time spent walking (≥1, 0.5–1 or <0.5 h/day, or missing), sleep duration (≤6, 7–8 or ≥9 h/day, or missing), education level (≤15, 16–18 or ≥19 years, or missing), history of disease (stroke, hypertension, myocardial infarction, cancer or diabetes [yes or no for each term]), cognitive function score (0, ≥1 or missing) and social participation (volunteering, hobby activities, or activities in neighborhood association [yes or no for each term]).

CI, confidence interval; OR, odds ratio; Ref., referent values.

## Discussion

To our knowledge, this is the first study to examine the association between the number of remaining teeth and healthy aging. When 0–9 teeth was used as the reference category, the category “10–19 teeth” was not statistically significant, but the categories “20–24 teeth” and “≥25 teeth” were significantly associated with healthy aging, regardless of whether they used dentures. These results suggest that having ≥20 natural teeth in older age is important to achieve healthy aging. The present findings would serve as evidence for the public health goal, such as Global Goals for Oral Health 2020 ^11^ and Health Japan 21.^12^

Previous studies have reported that maintaining more natural teeth is significantly associated with survival,^23^ independence in carrying out activities of daily living,^6^ better mental health^9^ and a high quality of life.^24^ These findings were consistent with those in the present study. Having a sufficient number of teeth helps maintains the chewing function, leading pleasurable eating of a variety of foods and taking enough nutrition.^25^ In addition, in terms of aesthetics, having enough teeth has been suggested as being essential for actively talking and laughing.^26^, ^27^ From the aforementioned, it has been clarified that a sufficient number of teeth affects not only the physical aspect, but also the mental aspect. Therefore, the number of teeth could be a predictor of healthy aging, including happiness, satisfaction and fulfillment advocated by the World Health Organization.

In Japan, the 8020 Campaign advocating to maintain ≥20 teeth until the age of 80 years has been widespread across the nation, and the percentage of individuals maintaining ≥20 remaining teeth until the age of 80 years has increased from 25.0% in 2005 to 51.2% in 2016.^28^ Globally, the prevalence of dental caries is decreasing, but periodontal disease is increasing.^29^ Therefore, to realize a healthy aging society on a global scale, strategies for improving oral health are indispensable.

The present study had a number of strengths. First, we used a relatively large population‐based cohort with 8300 older participants. Second, for our definition of healthy aging, we considered not only physiological, but also psychological indicators.

However, the present study also had several limitations. First, although we excluded participants with depressive symptoms, we could not obtain information about HRQOL and life satisfaction at baseline. Second, misclassification of the number of teeth as a result of self‐reporting might have occurred. However, the validity of the self‐reported number of teeth has been confirmed in a previous study.^30^

In conclusion, the results of the present study suggest that maintaining ≥20 teeth is associated with healthy aging in older people in Japan. This finding would serve as evidence for the public health goal of having older people around the world maintain ≥20 teeth to achieve healthy aging.

## Disclosure statement

The authors declare no conflict of interest.

## Supporting information


**Table S1** Baseline characteristics of the study participants according to healthy aging (*n* = 8300).

## Data Availability

Research data are not shared.
