# Supplementary material for: Association between number of remaining teeth and healthy aging in Japanese older people: The Ohsaki Cohort 2006 Study
Source: Geriatr Gerontol Int. 2021 Dec 1;22(1):68–74. doi: 10.1111/ggi.14320 (PMC9299646; doi:10.1111/ggi.14320)
Supplement: Supplementary file 1 — Table S1 Baseline characteristics of the study participants according to healthy aging (n = 8300). [file GGI-22-68-s001.docx]

| Supplementary Table 1. Baseline characteristics of the study participants according to healthy aging (n = 8300). | | | | | |
| --- | --- | --- | --- | --- | --- |
|  | Healthy aging | | Non-healthy aging | |  |
|  | (n = 621) | | (n = 7679) | | *P*-values |
| Age (years) (mean (SD)) | 69.5 (3.8) | | 73.6 (5.7) | | < 0.001 |
| Sex (%) |  |  |  |  |  |
| Men | 55.2 | | 47.8 | | < 0.001 |
| Women | 44.8 | | 52.2 | |  |
| Number of remaining teeth (%) |  | |  | |  |
| 0–9 | 24.8 | | 42.4 | | < 0.001 |
| 10–19 | 21.1 | | 24.6 | |  |
| 20–24 | 27.5 | | 19.3 | |  |
| ≥25 | 26.6 | | 13.7 | |  |
| Body mass index (kg/m^2^) (%) |  | |  | |  |
| ≤18.4 | 2.1 | | 4.3 | | 0.011 |
| 18.5–24.9 | 69.7 | | 65.2 | |  |
| ≥25.0 | 28.2 | | 30.5 | |  |
| Smoking status (%) |  | |  | |  |
| Current | 12.9 | | 13.4 | | < 0.800 |
| Former | 29.1 | | 27.9 | |  |
| Never | 58.0 | | 58.7 | |  |
| Drinking status (%) |  | |  | |  |
| Current | 51.8 | | 40.9 | | < 0.001 |
| Former | 5.9 | | 10.1 | |  |
| Never | 42.3 | | 49.0 | |  |
| Time spent walking (h/d) (%) | |  |  |  |  |
| ≥1.0 | 35.9 | | 30.1 | | < 0.001 |
| 0.5–1.0 | 42.2 | | 38.7 | |  |
| <0.5 | 21.9 | | 31.2 | |  |
| Sleep duration (h/d) (%) |  |  |  |  |  |
| ≤6 | 16.9 | | 19.0 | | < 0.001 |
| 7–8 | 70.3 | | 60.2 | |  |
| ≥9 | 12.8 | | 20.8 | |  |
| Education level ^†^ (%) | | |  |  |  |
| ≤15 years | 14.7 | | 27.4 | | < 0.001 |
| 16-18 years | 48.0 | | 43.9 | |  |
| ≥19 years | 37.3 | | 28.7 | |  |
| History of disease (%) |  |  |  |  |  |
| Stroke | 1.5 | | 2.3 | | 0.166 |
| Hypertension | 35.8 | | 43.3 | | < 0.001 |
| Myocardial infarction | 1.5 | | 4.4 | | < 0.001 |
| Diabetes mellitus | 8.2 | | 11.1 | | 0.027 |
| Cancer | 6.4 | | 8.4 | | 0.094 |
| Better cognitive function ^‡^ (%) | 82.5 | | 70.5 | | < 0.001 |
| Social participation (%) |  |  |  |  |  |
| Volunteering | 56.8 | | 36.1 | | < 0.001 |
| Hobby activities | 74.6 | | 52.0 | | < 0.001 |
| Activities in neighborhood association | 68.6 | | 53.3 | | < 0.001 |
| We used χ^2^ test for variables of proportion and one-factor ANOVA for continuous variables (missing value excluded). | | | | | |
| ^†^ Age at last school graduation. | | | | | |
| ^‡^ Cognitive function score in Kihon Checklist <1. | | | | | |
